# Malunion of a Clavicle Fracture in a Young Adult: A Case Report and Surgical Intervention

**DOI:** 10.7759/cureus.48202

**Published:** 2023-11-03

**Authors:** Abhiram A Sahasrabhojanee, Ashok M Mehendale, Dhananjay Gupta, Pranav Gupta, Gauri Kakar

**Affiliations:** 1 Medicine and Surgery, Jawaharlal Nehru Medical College, Datta Meghe Institute of Higher Education and Research, Wardha, IND; 2 Preventive and Social Medicine, Jawaharlal Nehru Medical College, Datta Meghe Institute of Higher Education and Research, Wardha, IND; 3 Orthopedics, Fortis Flt. Lt. Rajan Dhall Hospital, New Delhi, IND

**Keywords:** shoulder, fracture, rehabilitation, malunion, clavicle

## Abstract

This case study examines the medical complexity of managing a neglected clavicle in a young patient, resulting in a complicated interstitial non-union. Despite initial therapeutic efforts, the fracture was not treated, resulting in significant pain as well as functional and aesthetic disabilities. This case outlines accurate clinical presentation and diagnostic methods. In addition, malunion clavicle fractures require multifaceted therapeutic approaches including surgical interventions, rehabilitation programs, and psychological support. Through comprehensive research and long-term follow-up, this report reveals the complexity of traumatic fractures, highlighting the importance of early recognition and intervention. To address the issue effectively, it is essential to follow a multidisciplinary approach that includes physical assessment, pharmacotherapy, and physiotherapy. This case report aims to highlight the critical role of comprehensive individual care in improving the patient’s condition and emphasizes the importance of vigilant healthcare practices.

## Introduction

Around 5% to 10% of all fractures are clavicle fractures [1]. Clavicular non-union, a rare complication that can occur after non-operative treatment, has been documented in 0.1% to 24% of patients [2]. It can be incapacitating and typically manifests as shoulder pain and movement restrictions [3]. Clavicle shortening of less than 15-20 mm, female sex, fracture comminution, fracture displacement, advanced age, severe initial trauma, and unstable lateral fractures (Neer type II) are risk factors for clavicular non-union. According to Schnetzke et al.'s study of 58 patients, there were 33% atrophic, 20% hypertrophic, 7% mixed type, and 40% delayed fracture healing complications in the clavicle fracture healing complications category [4]. Non-unions with symptoms such as pain and functional shoulder impairment are typically surgically treated. The clavicle can be completely or partially removed surgically, and the clavicle can also be rebuilt. Clavicle resection has been abandoned in favor of implant technology for anatomic reconstruction. A bone union is achieved with fracture site reduction, stable fixation, and bone grafting transplantation from the site itself or the iliac crest at this time [5]. The general principles for treating clavicular non-unions are the same as those for other fracture sites. Fixation frequently occurs internally and needs to offer stability [5]. The use of bone grafts is advised by numerous authors to encourage local biology for healing. In the case of gap non-union, such as in our case, strut grafts such as tricortical grafts or fibula are a must to bridge the defect [6]. The purpose of this presentation is to highlight the importance of a multidisciplinary approach in the management of clavicular non-unions, emphasizing the various treatment options and factors influencing their occurrence and treatment outcomes.

## Case presentation

**Figure 1:**
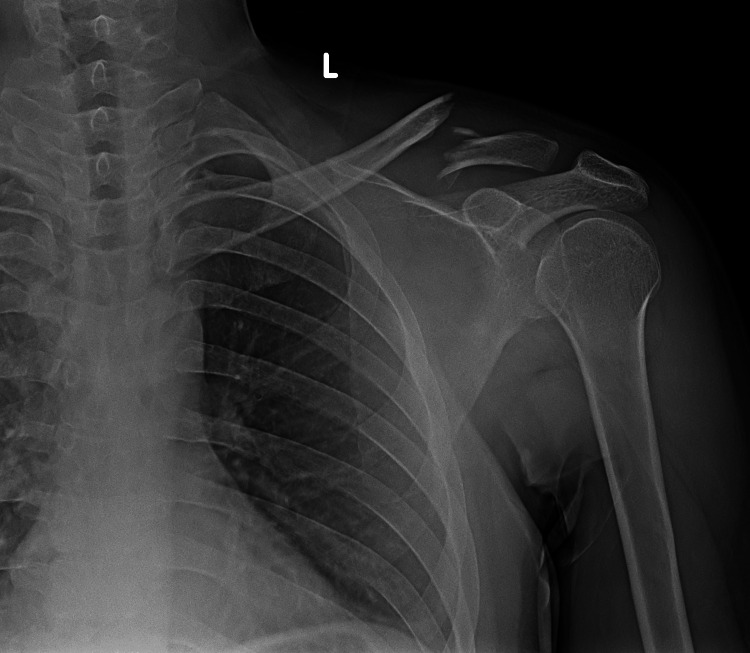
Initial X-ray scan showing a fractured clavicle

A female presented to the hospital with a displaced fracture of the clavicle, which led to a complication due to the autonomy of the patient to choose delayed treatment. The patient was advised to undergo surgery but refused for the same due to the family's disapproval of the scar marks associated with the surgery. Hence, the fracture was treated conservatively with a clavicle brace, and calcium and vitamin D supplements. The patient was prescribed vitamin D and calcium supplements due to the identification of low serum levels, as well as in consideration of the established role of vitamin D and calcium in facilitating expedited recovery processes. The brace was used for six weeks. Figure 1 shows the initial X-ray of the patient at the time of presentation. The fracture was expected to heal and union to take place. After a stipulated period, the brace was removed. The patient was advised to have shoulder movements. The patient continued to have pain over the fracture site, especially during shoulder movements or lifting weights. Moreover, the patient and her family also avoided further consultations. Almost a year after the incident, the patient complained of abnormal mobility at the fracture site along with weakness (grade 1) and slight discomfort in shoulder movements. The sensation of weakness was continuous along with pain. It was as severe as in an injured and exhausted patient.

**Figure 2:**
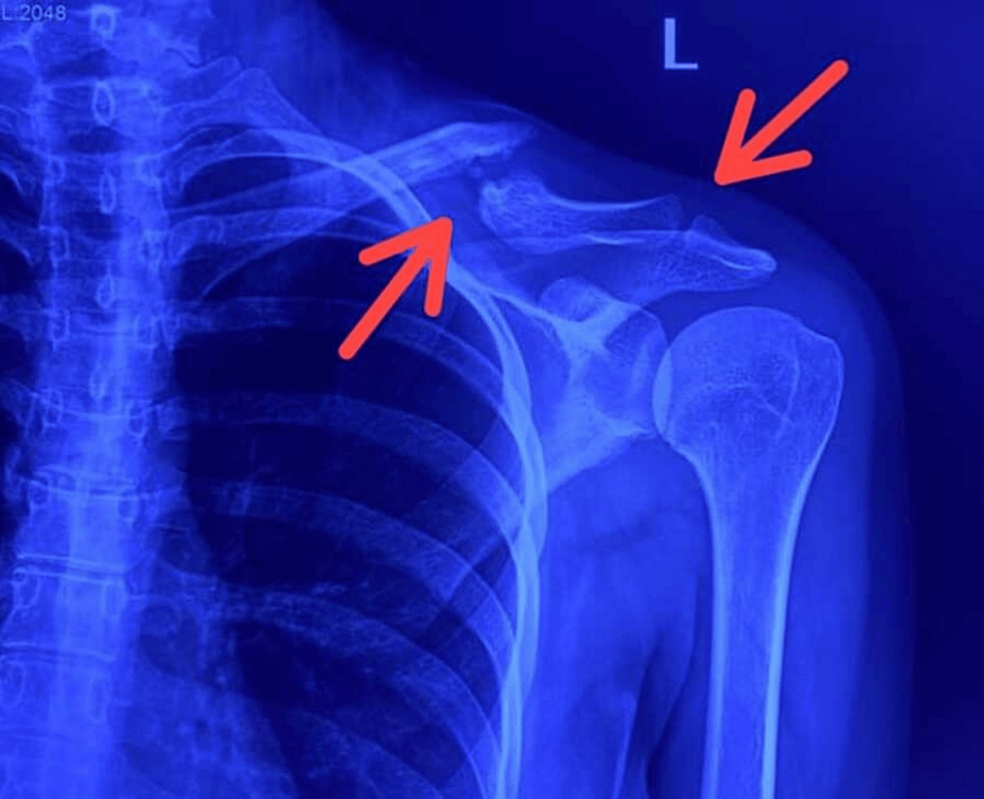
X-ray scan showing frank non-union with atrophic bone ends and gap between the ends Non-union with atrophic bone ends and gap between the ends (red arrows)

The pain was excruciating, acute, and aggravated by movement. The above symptoms were neglected by the family members. The patient continued with her supplements and physiotherapy. Three months later, the patient also developed swelling in the left clavicular region. The swelling was red, painful and not relieved by any medication. X-ray of the chest revealed frank non-union with atrophic bone ends and a gap between the ends. Malunion is diagnosed when the bone has healed in an abnormal position, resulting in functional limitations, deformity, or pain. Non-union, on the other hand, is diagnosed when the fracture fails to heal within the expected timeframe, as evidenced by the absence of bone healing on imaging and ongoing clinical symptoms (Figure 2). The patient was advised to undergo surgery yet again, but the family still did not agree to it. Eventually, after careful consideration and convincing, consent was obtained for surgery.

Physical examination

For further investigations, a comprehensive physical examination was conducted to assess the patient’s overall health focusing on the musculoskeletal concerns. Table 1 summarizes the key findings obtained through this examination, providing crucial insights into the patient’s clinical status and serving as a foundation for the subsequent diagnostic and therapeutic interventions. Local examination revealed gross muscle wasting of the shoulder and scapular regions, and frank abnormal mobility at the lateral third of the clavicle with mild tenderness. Painful terminal restriction of movements of the left shoulder was observed.

**Table 1:** Physical examination's findings

Findings	Description
General appearance	Age: 21 years; sex: female; well nourished
Vital signs	Blood pressure: 110/70 mmHg; heart rate: beats 82/minute; respiratory rate: 18 breaths/minute; temperature: 98.6 degrees Fahrenheit
Chest and lungs	Symmetrical chest expansion; breath sounds clearly bilateral; no wheezing, rales, rhonchi
Cardiovascular examination	Regular rhythm; S1 and S2 were heard; no murmurs, rubs, gallops; peripheral pulses present and equal
Abdomen	Soft and non-tender; no palpable masses; bowel sounds present
Extremities	No pallor, icterus, clubbing, cyanosis, lymphadenopathy, or edema present
Central nervous system	Alert and oriented to time, place, and person; cranial nerves intact; no abnormal reflexes
Local examination	Range of motion of the left shoulder: painful and restricted; tenderness present at the midclavicular region on the left side; crepitus present

Surgery notes

**Figure 3:**
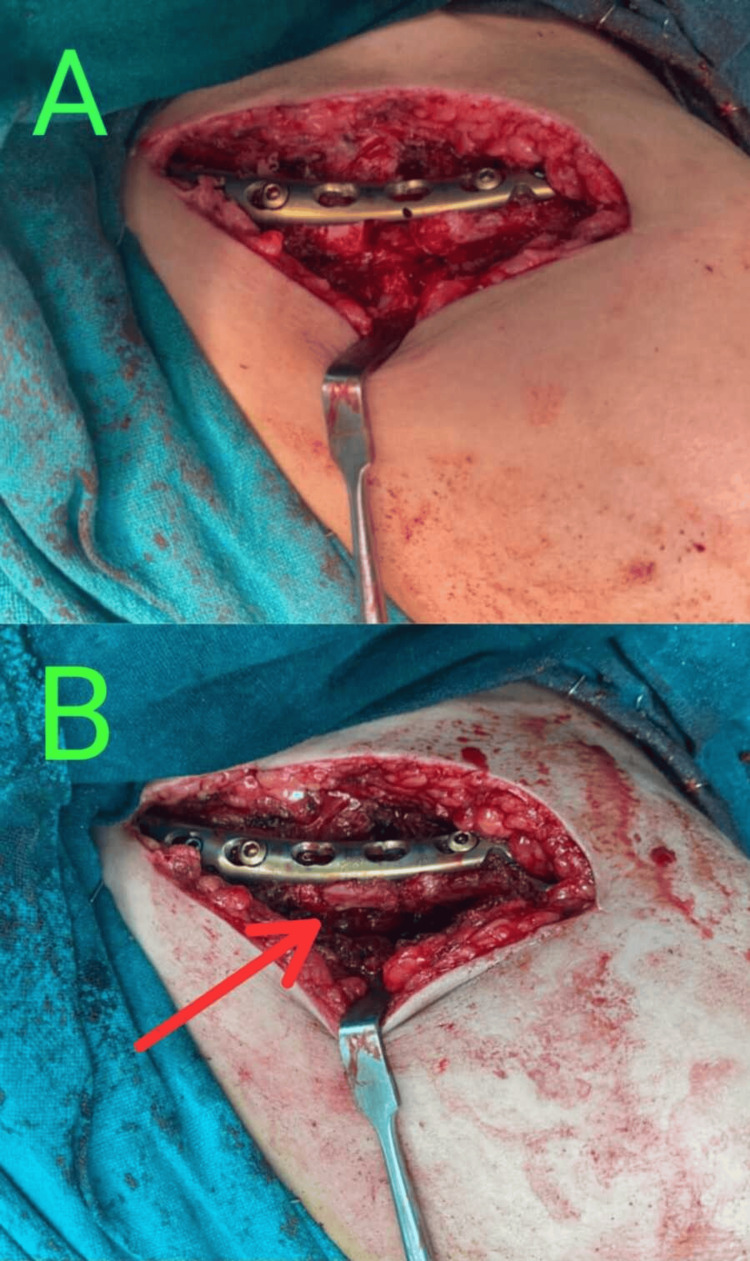
Intraoperative image showcasing clavicle fracture fixation (A) Stabilization using a recontoured clavicle plate. (B) Tricortical bone grafting from the ipsilateral iliac crest to bridge the gap between the fracture ends (red arrow).

The patient was operated on after obtaining informed consent and anesthetic clearance. The left shoulder and the left iliac crest were cleansed and draped after putting a sandbag under the left scapula and the left buttock. The patient underwent open reduction and internal fixation (ORIF) of the left clavicle with bone grafting under general anesthesia (GA) and supraclavicular block. The surgeon made sure that there was wide exposure via a wide anterosuperior approach preserving cutaneous nerves leading to exposure of the fracture site followed by the excision of interposing fibrous tissue. Nibbling of bone ends was performed until the edges were visible. An intramedullary canal was opened on both sides to establish an endosteal blood supply. A gap of around 3 cm was created after freshening of fractured edges. The fracture was stabilized with a pre-contoured 8-hole 3.5 titanium plate (Synthes Johnson &Johnson, Davos, Switzerland), as shown in Figure 3.

**Figure 4:**
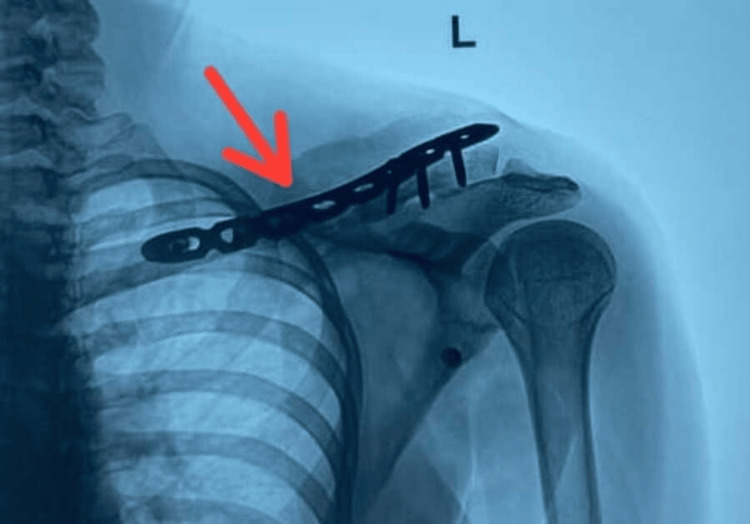
Postoperative radiograph Stabilization using a recontoured clavicle plate (red arrow)

A tricortical bone graft was harvested from the ipsilateral iliac crest. The graft was appropriately shaped and wedged between the fracture ends. The surgeons could not pass the screw through it; hence, tied it with an absorbable suture. Bone chips were packed around the area via a subcuticular closure to minimize scarring. The arm was kept in an arm pouch for four weeks. Pendulum exercises were initiated from the first day. The shoulder was mobilized after three weeks. The patient tolerated the procedure well. The postoperative period was uneventful. Chest X-ray was satisfactory (Figure 4). Dressing was changed on the second postoperative day. The patient was ambulatory. At the three-month postoperative assessment, the patient's condition had significantly improved, as evidenced by a Disabilities of the Arm, Shoulder, and Hand (DASH) score of 1.81 on a scale of 0 to 20.68. Furthermore, promising signals of healing in the patient's clavicle fracture have been discovered. The patient is expected to make a full recovery with the help of continued rehabilitation and frequent follow-up care. The patient is now discharged and in a stable condition.

## Discussion

Cases of clavicle non-union present unique challenges and considerations for orthopedic surgeons. Clavicle non-union rates typically fall between 0.1% and 24%. Timing of acceptance and intervention are vital factors affecting the outcomes in these cases. One important point to mention is the selection of appropriate surgical techniques. In this regard, the decision to perform ORIF with plate screws was critical [7]. The ORIF provides a stable fixation, allowing for faster mobilization and reducing the risk of fracture. The choice of surgical technique, careful dissection, and precise reconstruction contributed immensely to the outcome [8].

The significance of post-operative rehabilitation should also be emphasized more during the conversation. Young adults regain not only function but also shoulder mobility and strength. Exercises that are part of a rehabilitation program aid in regaining range of motion, stability in the joints, and muscle strength. To track progress and make necessary modifications to the rehabilitation program, it is crucial to follow through with the prescribed exercises and to conduct routine follow-up checks. Additionally, it is important to address any psychological side effects of surgery [9]. A visible disability can cause self-esteem problems and social isolation in young adolescents because they are generally more self-conscious about their physical appearance. In addition to improving motor function, surgical correction also boosts patient confidence and overall quality of life. During recovery, counseling and assistance are crucial for the emotional aspects of the patient’s well-being [10].

Another important point to mention is the potential complications of interventional surgery. Infection, hardware failure, and muscle or tissue damage are risks that must be managed with caution. Aggressive infection control strategies, close monitoring of infection symptoms, and informed consent with emphasis on potential risks and complications are important aspects of surgery Finally, it is important to consider what the duration of young adult swans will be [11]. Properly managed data, such as the announcement, tend to produce better results in the long run. However, continued follow-up is necessary to monitor for late complications, such as hardware issues or delayed treatment, and to ensure that functional improvement is maintained over time. In summary, the discussion should encompass a variety of factors, including surgical technique, post-operative rehabilitation, psychological effects, complications, and long-term considerations. Understanding these aspects will aid in the better management of clavicle malunion cases in the near future [12].

## Conclusions

In conclusion, this case study underscores the timely and appropriate management in addressing the malunion of clavicular fractures in young adults. Through a comprehensive assessment and surgical intervention, we were able to achieve a successful outcome, emphasizing the importance of individualized treatment plans tailored to the patient’s unique clinical presentation. Overlooked clavicle fractures in younger patients pose significant therapeutic complications. Timely intervention, whether conservative or surgical, is essential to prevent complications and ensure a good functional outcome. This case report highlights the challenges associated with neglected clavicle fractures and emphasizes the importance of proper management to improve the quality of life of affected individuals. This report serves as a valuable contribution to the understanding and management of clavicular fractures and their potential complications in the young adult population, highlighting the necessity of multidisciplinary collaboration in optimizing patient care and promoting favorable long-term outcomes.
